# Association of stroke and bleed events in non-valvular atrial fibrillation patients with direct oral anticoagulant prescriptions in NHS England between 2013 and 2016

**DOI:** 10.1371/journal.pone.0218878

**Published:** 2019-06-24

**Authors:** Harsh Sheth, Daniel McNally, Mauro Santibanez-Koref, John Burn

**Affiliations:** 1 Institute of Genetic Medicine, Newcastle University, Newcastle upon Tyne, United Kingdom; 2 Foundation for Research in Genetics and Endocrinology, Institute of Human Genetics, Ahmedabad, Gujarat, India; Indiana University, UNITED STATES

## Abstract

Prescription of direct oral anticoagulants (DOAC) compared to warfarin for treating atrial fibrillation patients have increased substantially since their introduction in the England’s National Health Service. Assessment of the risk of strokes and bleeds in relation to the large-scale uptake in DOACs compared to warfarin at the clinical commissioning group (CCG) level needs to be carried out. Publicly available- aggregated, CCG level, multi-source health and prescription records data were interrogated to investigate the association between prescription rate of DOACs and stroke/ bleed events during the period of 2013 to 2016. Variability of prescription rates and patient numbers across 208 CCGs were used to infer the effect of DOACs on stroke and bleed risk. Relative risk (RR) and 95% credible intervals (CI) were estimated using Markov chain Monte Carlo approach in JAGS. During the study period, the proportion of DOAC prescriptions increased at an average rate of 122% per annum. DOAC prescription was association with a 50% reduction in ischaemic (RR = 0.48, 95% CI = 0.39, 0.57) and haemorrhagic stroke (RR = 0.50, 95% CI = 0.26–0.77). In contrast, DOAC prescription reached significant association with reduction in gastrointestinal bleeds (RR = 0.86, 95% CI = 0.73–0.98) but not clinically relevant bleeds (RR = 0.95, 95% CI = 0.85–1.05). Sex stratified data showed significant association between DOAC prescription and reduction in haemorrhagic stroke risk (RR = 0.40, 95% CI = 0.28–0.52) and gastrointestinal bleeds (RR = 0.76, 95% CI = 0.63–0.93) in males only. Age stratified data suggested significant association with reduction in risk of both ischaemic and haemorrhagic strokes in patients aged 70 years and above, and reduction in risk of clinically relevant and gastrointestinal bleeds in patients aged 70–79 years only. Publicly available health and prescription data for the English population indicates reduction in stroke and bleed risk in specific age and sex sub-groups with the uptake of DOACs compared to warfarin between 2013 and 2016.

## Introduction

It is estimated that approximately 1.4 million people in England, which is equal to 2.5% of the population, have atrial fibrillation (AF) [1]. Antiplatelet agents are deemed ineffective at reducing risk of stroke in AF patients [2]; thereby necessitating long-term anticoagulation using alternative medications. Warfarin has been the mainstay of anticoagulation for the better part of the last 60 years. However, in the past 8 years, its use has been gradually replaced by a new class of anticoagulants called direct oral anticoagulants (DOACs) that includes dabigatran, rivaroxaban, apixaban and edoxaban. Unlike warfarin, DOACs have a wider therapeutic window, can be prescribed at set doses, have faster onset and offset of action and generally do not require patient monitoring for the degree of anticoagulation. Due to these attributes, DOAC prescriptions have increased dramatically in the UK primary care with them accounting for approximately 56% of all first-time oral anticoagulant prescriptions [3].

A series of large-scale randomized controlled trials (RCT) and subsequent meta-analysis of these trials have established non-inferiority of DOACs for stroke prevention and superiority for reducing haemorrhagic events in AF patients, compared to warfarin [4–8]. However, there have been residual concerns about the safety and efficacy of DOACs compared to warfarin in the real world scenario, where a broad range of patients are treated with anticoagulants. Furthermore, a major uptake of DOACs in clinical practice in the UK has put a significant burden on the healthcare budget; expenditure of anticoagulants rose by over a £100 million in 2016 with the estimate for the costs to rise to £1 billion per year by 2020 [9]. Lastly, a recent analysis of adherence in England based on a representative review of repeat prescription issuance suggests poor adherence with DOACs compared to warfarin [9]. A similar result has been reported in Canada where approximately one in three patients were non-persistent to dabigatran and rivaroxaban within 6 months from initiation [10]. Both results could be attributed to the lack of routine monitoring requirement for DOACs in contrast to warfarin.

Despite these concerns, a prospective patient-level study by Green *et al*. in the English general population from 2013–2016 showed that patients on DOACs were associated with lower odds of an intracranial haemorrhage versus gastrointestinal (GI) bleeding, compared to warfarin [11]. A more in-depth patient-level prospective study by Vinogradova *et al*. in the English population between 2011 and 2016 showed that apixaban was the safest drug amongst the DOACs compared to warfarin, with reduced risk of clinically relevant bleeding, intracranial bleeding and GI bleeding [12]. Together, these results suggest concordance of safety results between the RCTs and the real world population at patient-level. However, it is yet unclear whether the mass uptake of DOACs led to a significant improvement in stroke and bleed rates in England.

Following the re-organisation of the England’s National Health Service (NHS) in 2012, decisions on routine clinical policies have been determined at individual clinical commissioning group (CCG) level in England, with each responsible for approximately 100,000–900,000 people [13]. This offered a natural experiment to assess the association between DOAC prescription and risk of stroke/ bleed events in AF patients at the CCG level between 2013 and 2016, in order to detect risk-benefit of large scale uptake in DOAC prescription. We used aggregated, CCG level, multi-source health records data to investigate these associations and provide separate results for strokes and bleeds, with further stratification by sex and age. We used a Bayesian approach whereby, variability of the aggregate number of DOAC prescriptions and aggregate number of stroke/ bleed events across 208 CCGs was used to infer the association between DOACs versus warfarin prescription and stroke/ bleed risk.

## Materials and methods

### Eligibility and data collection

**Fig 1:**
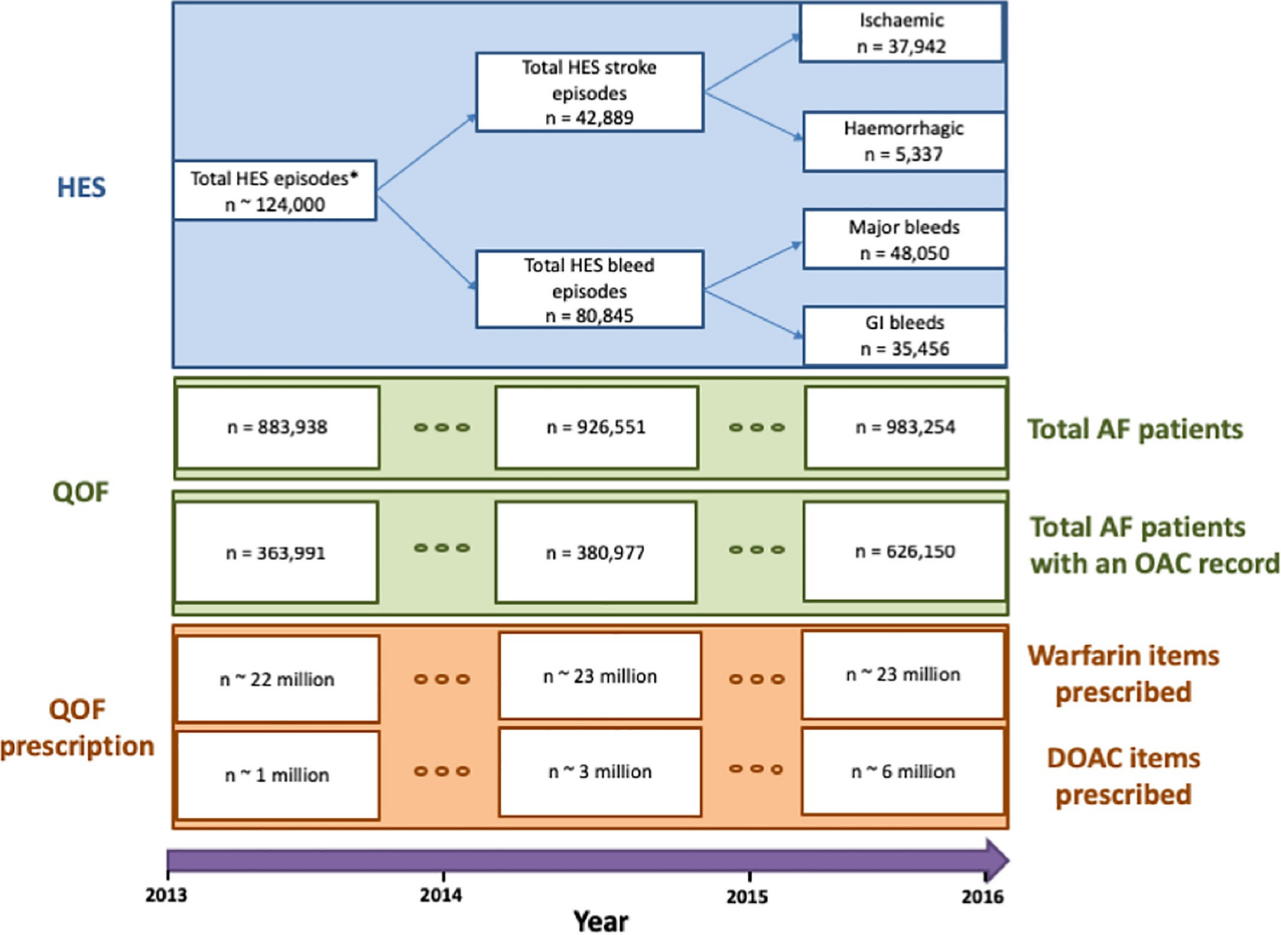
STROBE diagram depicting analytical cohorts and their associated time frames. HES data indicates the total number of hospital episodes of AF patients on long term oral anticoagulation with either a stroke or a bleed between 2013 and 2016 across all CCGs in England. QOF data indicates the total number of AF patients and the total number of AF patients on an oral anticoagulation treatment in primary care for a given year across all CCGs. QOF prescription data indicates the total number of warfarin and DOAC prescriptions made across all CCGs for a given year. HES = Hospital Episode Statistics, QOF = Quality Outcomes Framework, AF = Atrial fibrillation, DOAC = direct oral anticoagulant, OAC = oral anticoagulation. * Total episodes consisting of stroke and bleed episodes in AF patients on long term anticoagulation in the Hospital Episode Statistics database between 2013 and 2016.

A comprehensive set of aggregated English national data was compiled from various databases for the duration of 2013 to 2016, since no single database exists which contains all relevant information (Fig 1).

CCG level aggregate number of episodes of hospitalised non valvular AF-related stroke in England were accessed from the Hospital Episodes Statistics (HES) database (https://digital.nhs.uk/data-and-information/data-tools-and-services/data-services/hospital-episode-statistics). HES database contains details of hospital admissions, outpatient appointments and accidents and emergency attendances at the NHS England hospitals. Aggregated yearly counts of patients diagnosed with non-valvular AF related stroke or bleed (AF codes and hospital admission ICD-10 codes in S1 Table), who were on an anticoagulation treatment at the time of hospital admission (ICD-10 code: Z92.1) or had a clinical history of an anticoagulation treatment before the time of admission (ICD-10 code: Y44.2) were extracted from HES data between 2013 and 2016. Aggregate counts were further stratified by stroke type (ischaemic and haemorrhagic), bleed type (gastrointestinal (GI) and clinically relevant), age groups (<60, 60–69, 70–79, 80–89 and ≥90 years at the time of admission) and sex (male or female).

The English CCG level aggregate number of AF patients (AF001 code) and number of AF patients on an oral anticoagulation were derived from the Clinical Domain of the Quality and Outcomes Framework (QOF) database (https://qof.digital.nhs.uk) for the period of 2013 to 2016. QOF is a national primary care database covering all primary care practices in England and collects prevalence and treatment data on numerous conditions, including AF. CCG-level annual summary concerning the uptake of oral anticoagulants for patients residing in England with a moderate to high risk of stroke measured either with CHADS2 score≥1 for the period 2013–2015 and CHA2DS2-VASc score≥2 for the period 2015–2016 were obtained from the QOF database. Furthermore, annual aggregate number of warfarin and DOAC prescriptions made at the primary care level within each CCG were also derived from QOF database. Lastly, CCG level aggregate number of AF patients and number of patients an on oral anticoagulant were stratified into age groups and sex strata with data available from The Health Improvement Network (THIN) database (http://www.ucl.ac.uk/pcph/research/thin-database/database). THIN database collects primary care electronic health records of 11.1 million patients from 562 general practices in the UK. The database houses anonymised patient level medical diagnoses and treatment data, including AF and oral anticoagulant prescription. Estimates of the number of patients in each strata for each CCG were derived by multiplying the aggregate number of patients in the QOF database with the proportion of patients in a given strata from THIN database (Stratification methodology in S1 Table).

Ethics approval was not required for the current study as the data is publicly available for analysis by the NHS Digital. All raw data is available in S1 File.

### Data synthesis and statistical analysis

CCGs where the aggregate value for stroke or bleed event within any sub-group was 5*, indicated that there were fewer than 5 events in that sub-group within a given year. Access to the true number of events for these sub-groups was not available due to the risk of patient identification, hence, the values for these CCGs were rounded off to 5.

Pairwise comparison of mean values of all CCGs across the 3 years was carried out using two-sided Wilcoxon signed rank sum test. The relationship between the total number of AF patients on anticoagulation and stroke or bleed rates for all CCGs between 2013 and 2016 were modelled using non-parametric local-linear regression using Gaussian kernel and 300 bootstrap replications; using the -npregress- package in Stata v15. Effect size output from the non-parametric regression was presented as average marginal effect (as detailed in [14]). Relationship was measured using Spearman’s rank correlation coefficient.

The relationship between DOAC prescription and stroke or bleed risk, was assessed using a Markov chain Monte Carlo (MCMC) approach implemented in JAGS version 4.3.0 [15]. MCMC approach was utilised due to the availability of only CCG level summary stroke/ bleed event and anticoagulant prescription data rather than a patient-level data. In the absence of patient level data, MCMC, a Bayesian inference approach is useful as it allows to approximate aspects of posterior distributions that otherwise cannot be directly calculated. One critical assumption was made for the model used in the MCMC analysis: the probability of an age or sex stratified patient being treated with a DOAC was equal to the probability of an unstratified patient being treated with a DOAC within a given CCG, in the period of interest (MCMC codes included in S2 File). A total of 12,500 iterations, which included 2,500 iterations for burn-in, were used for each analysis. The results were presented as relative risk (RR) and 95% credible intervals (CI). We refer to the posterior credible interval overlapping RR value of 1 as non-significant. MCMC analyses were carried out for the overall dataset as well as age and sex stratified datasets. Data organisation was carried out in Stata v15 (Stata Corp, Texas, USA) and JAGS was run in R programming environment v3.5.1 (https://cran.r-project.org/).

## Results

### Study population

The present study analysed CCG level summary stroke, bleeds and oral anticoagulation prescription data from 208 CCGs in England, UK, across 2013 to 2016 (Table 1). Throughout the 3 years, there was an overall increase in the number of AF patients on anticoagulation (P <0.0001), with the largest increase observed in 2015–2016. Concurrently, there was an increase in the number of anticoagulation prescriptions, with the mean proportion of DOAC prescriptions increasing from 4.4% to 21.4% during this period (P <0.0001), suggesting an average increase in the proportion of DOAC prescription by 122% per annum. Whilst the overall number of strokes and bleeds have increased, the rate of strokes and bleeds per 1000 AF patients on anticoagulation have decreased during this period suggesting an inverse relationship between number of people on anticoagulation and stroke/ bleed rates (Stroke rate average marginal effect = -0.004 (-0.005, -0.002), p<0.001; Bleed rate average marginal effect = -0.006 (-0.007, -0.005), p<0.001; S1 Fig and S2 Fig).

**Table 1:** Summary statistics of the study population.

	Year			P-value^g^
	2013–2014	2014–2015	2015–2016	
**Number of CCGs** * **(n)**	209	209	209	-
**Total AF patients** ^**a**^ **(mean, SD per CCG)**	4213 (2704)	4417 (2844)	4704 (3014)	<0.0001
**AF patients on anticoagulation** ^**b**^ **(mean, SD per CCG)**	1734 (1120)	1815 (1153)	2995 (1938)	<0.0001
**Total number of anticoagulant prescriptions** ^**c**^ **(mean, SD per CCG)**	113318 (87073)	125875 (96336)	140040 (105933)	<0.0001
**Proportion of warfarin prescriptions** ^**d**^ **(mean, SD per CCG)**	95.6 (4.6)	89.3 (7.5)	78.6 (9.6)	<0.0001
**Proportion of DOAC prescriptions** ^**d**^ **(mean, SD per CCG)**	4.4 (4.6)	10.7 (7.5)	21.4 (9.6)	<0.0001
**Number of All strokes** ^**e**^ **(mean, SD per CCG)**	66.9 (43.6)	67.5 (42.5)	71.3 (44.4)	0.29^h^ and <0.0001
**Number of Ischaemic strokes (mean, SD per CCG)**	59.9 (39.4)	59.6 (37.9)	62.6 (39.4)	0.95^h^ and 0.004
**Number of Haemorrhagic strokes** **(mean, SD per CCG)**	8.3 (5.2)	9.0 (5.6)	9.7 (6.2)	<0.003
**Total number of All bleeds**^**f**^ **(mean, SD per CCG)**	117.2 (73.8)	127.9 (80.5)	141.9 (89.1)	<0.0001
**Number of Clinically relevant bleeds** **(mean, SD per CCG)**	70.4 (46.1)	75.6 (49.3)	83.9 (53.7)	<0.0001
**Number of GI bleeds** **(mean, SD per CCG)**	50.4 (32.1)	56.4 (36.3)	62.8 (40.1)	<0.0001

* Number of CCGs included in the final analysis was 208; Newcastle Gateshead CCG was not present in the year 2013–2014.

^a^ Aggregate number of patients registered at the GP practice with non-valvular atrial fibrillation (AF001 code) in the QOF database

^b^ Aggregate number of patients with CHADS2 score of 1 or above (AF004) on anticoagulation therapy in 2013–2015 and number of patients with CHA2DS2-VASc score of 2 or above (AF007) on anticoagulation therapy in 2015–2016. Data was obtained from QOF database.

^c^ Aggregate number of oral anticoagulant prescription made in primary care. Data was obtained from QOF database.

^d^ Proportion of the aggregate number of oral anticoagulant prescriptions that are either warfarin or DOACs. Data was obtained from QOF database.

^e^ Sum of the aggregate number of ischaemic and haemorrhagic strokes data available in HES database.

^f^ Sum of the aggregate number of clinically relevant and gastrointestinal bleeds data available in HES database.

^g^ P-value calculated using pairwise Wilcoxon signed rank test

^h^ P-value for pairwise Wilcoxon signed rank test for values from year 2013–2014 and 2014–2015.

CCG = Clinical Commissioning Groups, AF = Atrial Fibrillation, DOAC = Direct acting Oral Anticoagulants, SD = Standard Deviation, GI = Gastrointestinal, HES = Hospital Episodes Statistics, QOF = Quality Outcomes Framework.

### Association between DOAC prescription and stroke risk

**Fig 2:**
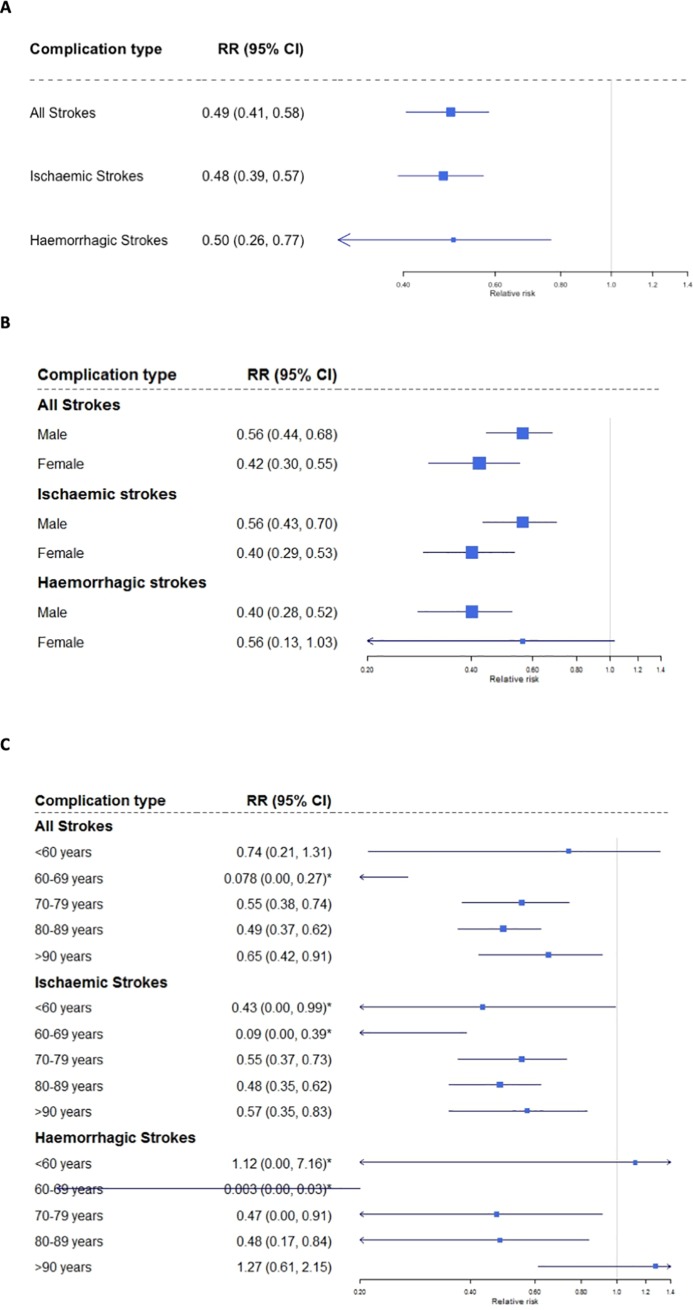
**Association between DOAC versus warfarin prescription and (A) overall stroke risk, (B) sex stratified stroke risk and (C) age stratified stroke risk.** RR = relative risk, CI = credible intervals. * Sub-group estimate with high autocorrelation within the MCMC model.

Analysis across all 3 years showed a 50% reduction in risk of overall stroke associated with DOAC prescription (RR = 0.49, 95% CI = 0.41–0.58; Fig 2A), compared with warfarin prescription. Stroke-type specific analysis showed approximately 50% decrease in both ischaemic stroke (RR = 0.48, 95% CI = 0.39, 0.57) and haemorrhagic stroke (RR = 0.50, 95% CI = 0.26–0.77) with DOAC prescription.

Females had lower risk of overall stroke and ischaemic stroke compared to males when prescribed a DOAC, compared to warfarin (Fig 2B). However, whilst males had 60% reduction in haemorrhagic stroke risk when prescribed a DOAC (RR = 0.40, 95% CI = 0.28–0.52; Fig 2B), a non-significant inverse association for risk reduction in females was observed (RR = 0.56, 95% CI = 0.13–1.03).

Interestingly, AF patients above the age of 70 years observed approximately 40–50% reduction in stroke risk (both ischaemic and haemorrhagic) when prescribed a DOAC compared to warfarin, with the exception of AF patients above 90 years of age who showed a trend for an increased risk of haemorrhagic strokes when prescribed a DOAC (Fig 2C). Relative risk for patients under the age of 70 years showed extremely large confidence intervals suggesting low variability in the prescription rates and patient numbers, which did not allow estimation of relative risk accurately.

### Association between DOAC prescription and bleed risk

**Fig 3:**
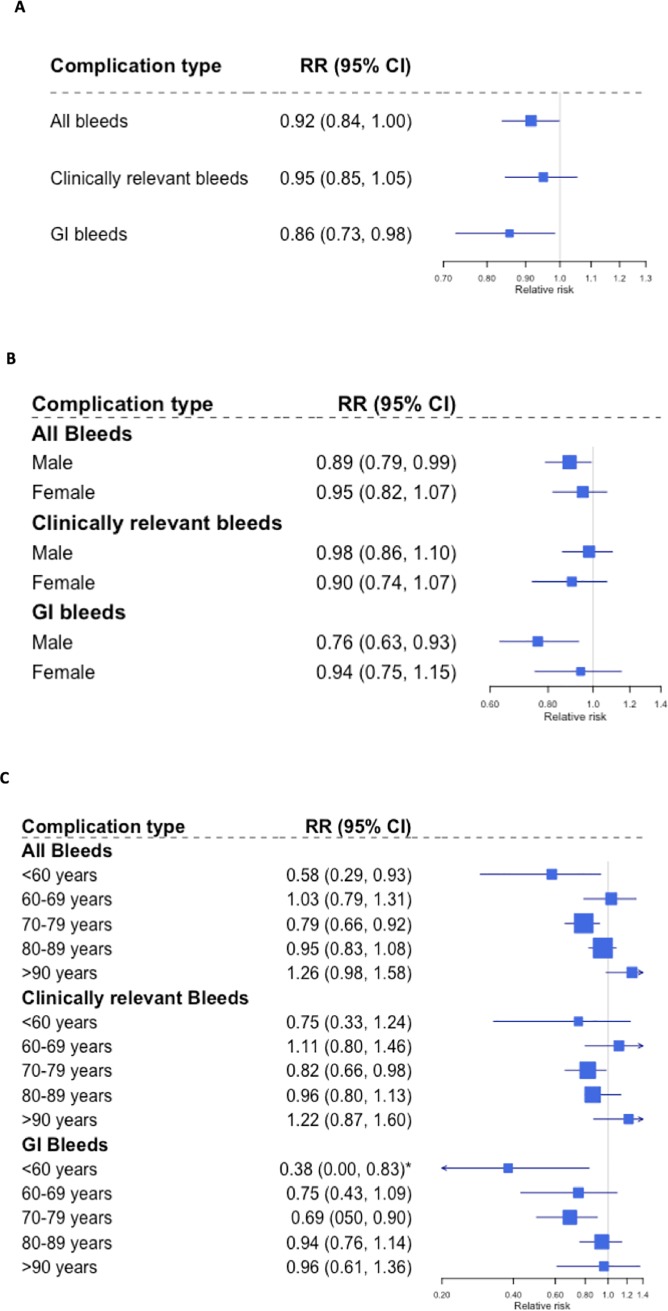
**Association between DOAC versus warfarin prescription and (A) overall bleed risk, (B) sex stratified bleed risk and (C) age stratified bleed risk.** RR = relative risk, CI = credible intervals. * Sub-group estimate with high autocorrelation within the MCMC model.

Unlike strokes, analysis across all 3 years showed a non-significant association between bleed risk and DOAC prescription compared to warfarin (All bleeds RR = 0.92, 95% CI = 0.84–1.00; Clinically relevant bleeds RR = 0.95, 95% CI = 0.85–1.05; Fig 3A). However, a modest decrease of approximately 14% was observed for GI bleed risk with DOAC prescription (RR = 0.86, 95% CI = 0.73–0.98).

Stratification by sex suggested approximately 10–20% decrease in all bleeds and GI bleeds risk for males with DOAC prescription compared to warfarin (All bleeds RR = 0.89, 95% CI = 0.79–0.99; GI bleeds RR = 0.76, 95% CI = 0.63–0.93; Fig 3B). In contrast, no risk reduction for all, clinically relevant or GI bleeds were observed for females with DOAC use (Fig 3B).

Lastly, stratification by age groups showed a reduced risk of bleeds in the age group 70–79 years only with DOAC prescription compared to warfarin (All bleeds RR = 0.79, 95% CI = 0.66–0.92; Clinically relevant bleeds RR = 0.82, 95% CI = 0.66–0.98; GI bleeds RR = 0.69, 95% CI = 0.50–0.90; Fig 3C). Interestingly, a trend for an increase in bleed risk was observed in patients above the age of 80 years with DOAC use, however, none of the associations were defined to be significant (Fig 3C).

## Discussion

In the present study, we aimed to assess association between DOAC versus warfarin prescription with stroke and bleed events, during the period of 2013–2016 in England’s National Health Service. Our analysis showed a 50% reduction in strokes, both ischaemic and haemorrhagic, with DOAC prescription compared to warfarin. However, a small but non-significant reduction in bleed risk, both clinically relevant and GI, was observed with DOAC prescription compared to warfarin. Stratification by sex and age showed stroke or bleed risk reduction in specific sub-groups compared to warfarin, in particular reduction in stroke risk for males and patients with age between 70–89 years, and reduction in bleed risk in patients with age between 70–79 years only.

Whilst an inverse association between haemorrhagic strokes and DOAC prescription compared with warfarin is congruent with the published trials and latest observational studies [4–7, 11, 12, 16], the inverse association of ischaemic stroke with DOAC prescription rate compared to warfarin is in contrast to the published results. This contrast however needs to be interpreted with caution. During the study period of 2013–2016, clinical guideline by the National Institute of Health and Care Excellence (NICE; CG180) in England recommended cessation of anti-platelet use for treating AF patients in order to prevent strokes [2]. This lead to a decrease in the use of anti-platelets for AF by 21.6% between 2011 and 2016 with a simultaneous increase in the use of DOACs from 0.1% in 2011 to 32.5% of all oral anticoagulation use in 2016 in England [17]. Interestingly, prescription trend data from the GRASP-AF (http://www.heartrhythmalliance.org/afa/uk/grasp-af), a tool used in primary care to assess risk of AF related stroke and efficacy of treatment, between 2009 and 2012 shows an age-dependent prescription of oral anticoagulants and antiplatelets, whereby for the CHADS_2_ scores of 1–6, the proportion prescribed an anticoagulant was lower in AF patients aged 80 and over than in those aged less than 80 years; a converse observation was made for antiplatelet prescription [18]. Hence, it is plausible that the introduction of NICE CG180 guidance would have steered a large proportion of patients aged 80 years and above, who have a higher CHADS_2_ score and hence are at a higher risk of stroke compared to patients aged below 80 [19], from an antiplatelet to an anticoagulant treatment, most likely DOACs, during the current study period. The switch in treatment would have likely reduced their risk of ischaemic stroke, the outcome of which being an inverse association between DOAC prescription and ischaemic stroke in the current study. However, assessment of this hypothesis is beyond the remit of the current study and should be examined in future studies.

Interestingly, sex stratified data suggests both males and females having an inverse association between ischaemic strokes and DOAC prescription, however, a statistically significant inverse association for haemorrhagic stroke with DOAC prescription is restricted to males only. The lack of significant association for females could be due to the lack of variability in the prescription rates and patient numbers; as can be observed with large credible intervals (see Fig 2B). A similar issue is observed in the age stratified stroke risk analysis for patients below the age of 70.

In contrast to strokes, DOAC prescription showed a small inverse but statistically non-significant association with bleeds. For bleed type stratified data, however, inverse association between DOAC prescription and GI bleeds reached significance. Furthermore, sex stratified bleed data suggested a significant inverse association between GI bleeds and DOAC use that is restricted to males only, in our study. This potentially novel observation could be explained by sex specific differences in the prescription of specific DOACs. Previous observational study and meta-analysis have shown inverse relationship between GI bleeds with apixaban and not rivaroxaban and dabigatran use in the AF patients [12, 16]. In the UK wide observational study by Green *et al*., approximately 50% of the dabigatran and rivaroxaban prescriptions were made to males, whereas, 61% of the apixaban prescriptions were made to males [11]. As apixaban has been associated with a lower risk of GI bleeds compared to warfarin, it is possible that the males may have derived greater benefit than females for reduction in GI bleeds. Lastly, age stratified data showed significant risk reduction for clinically relevant and GI bleeds to be associated with DOAC use in patients with age 70–79 years only. Beyond 79 years, there is a suggestion for a trend of an increase in bleed risk with DOAC use. This trend has previously been reported by Abraham *et al*., whereby they observed an increased risk of GI bleeds in AF patients with age more than 76 years, taking dabigatran (HR = 2.49, 95% CI = 1.61–3.83) or rivaroxaban (HR = 2.91, 95% CI = 1.65–4.81) compared to warfarin [20].

Of note, the code used in the QOF database which indicated the aggregate number of AF patients receiving an oral anticoagulation treatment changed from AF004 in 2013–2015 to AF007 in 2015–2016. The change in the code saw a corresponding minor increase in the proportion of moderate to high risk AF patients on anticoagulation from 84% in 2013–2015 to 87% in 2015–2016 but a large increase in the proportion of overall AF population that receives anticoagulation from 41% in 2013–2015 to 63% in 2015–2016. This substantial increase can be attributed to the ability of CHA2DS2-VASc score to enable identification of a larger number of AF patients for whom oral anticoagulation therapy is recommended compared to CHADS2 score [21]. Nonetheless, this wouldn’t have impacted the analysis as the analysis model required data on the moderate to high risk AF patients on oral anticoagulation rather than the overall number of AF patients in each CCG (see S2 File).

Whilst the current study has strengths such as systematic coverage of a single national health system, we acknowledge its limitations. First, the data were aggregated and not linked at the patient level, which precluded us from carrying out patient-level analysis and adjust for all possible confounding factors over the study period. This may potentially lead to our findings being incorrectly attributed or interpreted to the individual patient level. Second, our models assumed that DOAC prescription rate was same across all age and sex sub-groups for a given CCG and time period. In contrast to this, previous observational studies have shown that the probability of DOAC prescription differs between age-groups and sexes [11, 12]. Third, some of the CCGs where the stroke and/ or bleed events in specific sub-groups were less than 5 for a given year, the CCGs were coded as having 5 events. Knowledge of the true number of events was restricted to protect patient confidentiality. However, this approach may have reduced data variability, especially in certain age and sex subgroups. This may have not only reduced the accuracy of relative risk estimation but may have inflated risk estimates for certain subgroups. Fourth, whilst patients were selected based on the hospital codes describing them on either being on long term anticoagulation (ICD-10 code- Z92.1) and/ or having an adverse effect of anticoagulation medication in therapeutic use (ICD-10 code- Y44.2), it was not possible to gather detail on anticoagulation therapy duration. This could have implications on the bleeding risk, as the risk of bleeding is highest in the initial few months after initiation of anticoagulation therapy [22, 23].

In summary, our results suggest reduction in ischaemic and haemorrhagic stroke risk with increase in prescription of DOACs between 2013 and 2016 in England. Furthermore, a small but significant reduction in GI bleed risk is observed in specific subgroups, such as males and patients between 70–79 years of age, with increase in DOAC prescription.

## Supporting information

S1 Fig Correlation between number of people on anticoagulation and overall strokes per 1000 AF patients on anticoagulation across all CCGs between 2013 and 2016. Scatter plot depicting a non-linear inverse correlation between increase in number of people on anticoagulation and overall stroke rate. Stroke rate for each CCG was calculated by dividing aggregate number of strokes from the Hospital Episode Statistics database with aggregate number of AF patients on oral anticoagulation in primary care from the Quality and Outcomes Framework database (AF004 or AF007 code). AF = atrial fibrillation.
(TIFF)

S2 Fig Correlation between number of people on anticoagulation and all bleeds per 1000 AF patients on anticoagulation across all CCGs between 2013 and 2016. Scatter plot depicting a non-linear inverse correlation between increase in number of people on anticoagulation and all bleeds rate. Bleed rate for each CCG was calculated by dividing aggregate number of bleeds from the Hospital Episode Statistics database with aggregate number of AF patients on oral anticoagulation in primary care from the Quality and Outcomes Framework database (AF004 or AF007 code). AF = atrial fibrillation.
(TIFF)

S1 File Raw data from Hospital Episodes Statistics, Quality Outcomes Framework and The Health Improvement Network databases. (XLSX)

S2 File Markov Chain Monte Carlo simulation models for data analysis in JAGS. (DOCX)

S1 Table ICD-10 codes for atrial fibrillation, strokes, bleeds and anticoagulant drug description. (XLSX)
