# Supplementary figures and images for: Association of stroke and bleed events in non-valvular atrial fibrillation patients with direct oral anticoagulant prescriptions in NHS England between 2013 and 2016
Source: PLoS One. 2019 Jun 24;14(6):e0218878. doi: 10.1371/journal.pone.0218878 (PMC6590892; doi:10.1371/journal.pone.0218878)

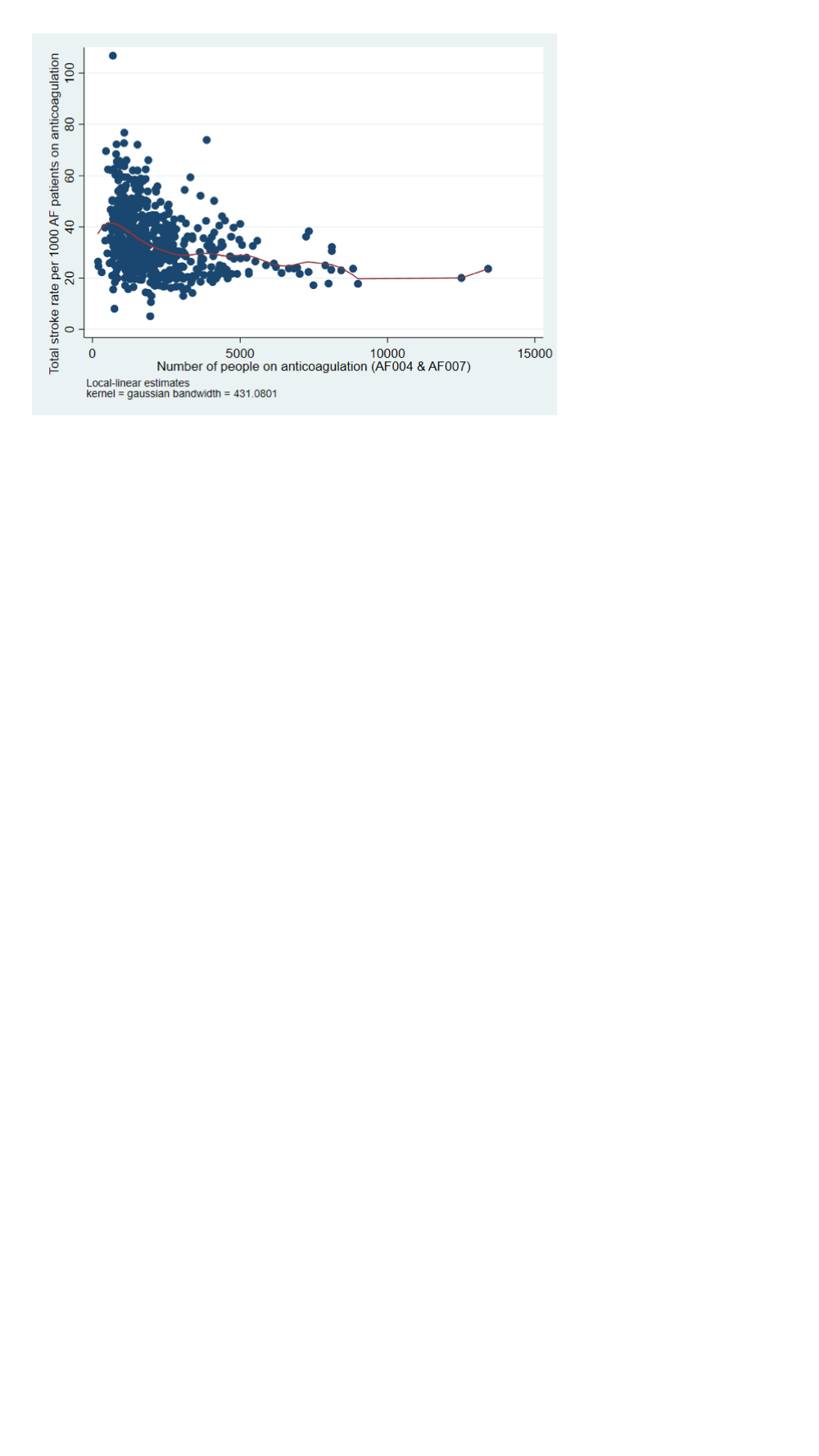

Supplement: S1 Fig — Scatter plot depicting a non-linear inverse correlation between increase in number of people on anticoagulation and overall stroke rate. Stroke rate for each CCG was calculated by dividing aggregate number of strokes from the Hospital Episode Statistics database with aggregate number of AF patients on oral anticoagulation in primary care from the Quality and Outcomes Framework database (AF004 or AF007 code). AF = atrial fibrillation. (TIFF) [file pone.0218878.s001.tiff]

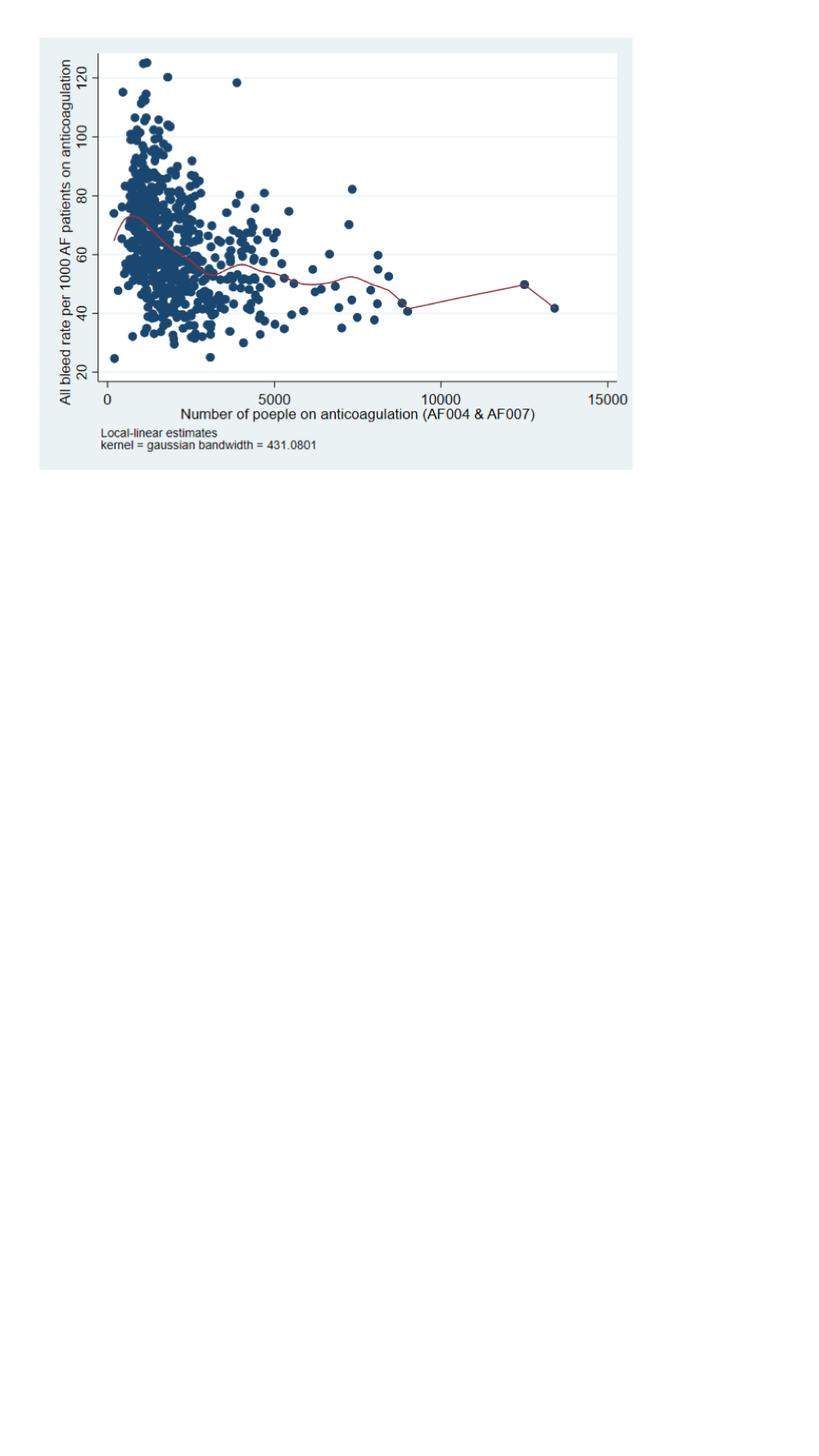

Supplement: S2 Fig — Scatter plot depicting a non-linear inverse correlation between increase in number of people on anticoagulation and all bleeds rate. Bleed rate for each CCG was calculated by dividing aggregate number of bleeds from the Hospital Episode Statistics database with aggregate number of AF patients on oral anticoagulation in primary care from the Quality and Outcomes Framework database (AF004 or AF007 code). AF = atrial fibrillation. (TIFF) [file pone.0218878.s002.tiff]
