# Supplementary material for: Association of stroke and bleed events in non-valvular atrial fibrillation patients with direct oral anticoagulant prescriptions in NHS England between 2013 and 2016
Source: PLoS One. 2019 Jun 24;14(6):e0218878. doi: 10.1371/journal.pone.0218878 (PMC6590892; doi:10.1371/journal.pone.0218878)
Supplement: S2 File — (DOCX) [file pone.0218878.s004.docx]

# Markov Chain Monte Carlo simulation model for overall data

**Designations:**

$p_{D}$: Probability of patient treated with a warfarin alternative in the period of interest

$p_{C}$: Probability of a patient suffering a complication in the period of interest

$p_{C*}$: Probability that a patient treated with warfarin develops a complication, i.e. $p\left( C|\bar{D} \right)$

$N$ : Total number of patients in the period of interest

$N_{C}$ : Total number of patients with complications in the period of interest

$N_{D}$ : Total number patient treated with warfarin alternative in the period of interest

$B\left( M,p \right)$ : The binomial distribution with parameters $M$ and $p$

**Assumption**

$$p\left( C|D \right)={\gamma p}_{C*}$$

Thus

$$p_{CD}=p\left( C|D \right)p_{D}={\gamma p}_{C*} p_{D}$$

$$p_{C\bar{D}}=p\left( C|\bar{D} \right)p_{\bar{D}}=p_{C*} \left( 1-p_{D} \right)$$

$$p_{\bar{C}D}=\left( 1-{\gamma p}_{C*} \right) p_{D}$$

$$p_{\bar{C}\bar{D}}=\left( 1-p_{C*} \right) \left( 1-p_{D} \right)$$

and for $p_{C}$ (the probability of complication)

$$p_{C}=p_{CD}+p_{C\bar{D}}={\gamma p}_{C*} p_{D}+p_{C*} \left( 1-p_{D} \right)= p_{C*}\left( \left( \gamma-1 \right)p_{D}+1 \right)$$

**Therefore:**

$$N_{D}\sim B\left( N,p_{D} \right)$$

$$N_{C}\sim B\left( N,p_{C} \right)$$

If we introduce the superscript $\left[ s \right]$to denote the period of interest, and assume that, the risk of complication remains the same across all periods:

$$N_{D}^{\left[ s \right]}\sim B\left( N^{\left[ s \right]},p_{D}^{\left[ s \right]} \right)$$

$$N_{C}^{\left[ s \right]}\sim B\left( N^{\left[ s \right]},p_{C}^{\left[ s \right]} \right)$$

$$p_{C}^{\left[ s \right]}= p_{C*}\left( \left( \gamma-1 \right)p_{D}^{\left[ s \right]}+1 \right)$$
